# Proton pencil minibeam irradiation of an *in-vivo* mouse ear model spares healthy tissue dependent on beam size

**DOI:** 10.1371/journal.pone.0224873

**Published:** 2019-11-25

**Authors:** Matthias Sammer, Esther Zahnbrecher, Sophie Dobiasch, Stefanie Girst, Christoph Greubel, Katarina Ilicic, Judith Reindl, Benjamin Schwarz, Christian Siebenwirth, Dietrich W. M. Walsh, Stephanie E. Combs, Günther Dollinger, Thomas E. Schmid

**Affiliations:** 1 Institut für Angewandte Physik und Messtechnik (LRT2), Universität der Bundeswehr München, Neubiberg, Germany; 2 Department of Radiation Oncology, Technical University of Munich, Klinikum rechts der Isar, Munich, Germany; 3 Institute of Radiation Medicine (IRM), Department of Radiation Sciences (DRS), Helmholtz Zentrum München (HMGU), Oberschleißheim, Germany; 4 Deutsches Konsortium für Translationale Krebsforschung (DKTK), Partner Site Munich, Germany; Central Research Institute of Electric Power Industry (CRIEPI), JAPAN

## Abstract

Proton radiotherapy using minibeams of sub-millimeter dimensions reduces side effects in comparison to conventional proton therapy due to spatial fractionation. Since the proton minibeams widen with depth, the homogeneous irradiation of a tumor can be ensured by adjusting the beam distances to tumor size and depth to maintain tumor control as in conventional proton therapy. The inherent advantages of protons in comparison to photons like a limited range that prevents a dosage of distal tissues are maintained by proton minibeams and can even be exploited for interlacing from different beam directions. A first animal study was conducted to systematically investigate and quantify the tissue-sparing effects of proton pencil minibeams as a function of beam size and dose distributions, using beam widths between σ = 95, 199, 306, 411, 561 and 883 μm (standard deviation) at a defined center-to-center beam distance (ctc) of 1.8 mm. The average dose of 60 Gy was distributed in 4x4 minibeams using 20 MeV protons (LET ~ 2.7 keV/μm). The induced radiation toxicities were measured by visible skin reactions and ear swelling for 90 days after irradiation. The largest applied beam size to ctc ratio (σ/ctc = 0.49) is similar to a homogeneous irradiation and leads to a significant 3-fold ear thickness increase compared to the control group. Erythema and desquamation was also increased significantly 3–4 weeks after irradiation. With decreasing beam sizes and thus decreasing σ/ctc, the maximum skin reactions are strongly reduced until no ear swelling or other visible skin reactions should occur for σ/ctc < 0.032 (extrapolated from data). These results demonstrate that proton pencil minibeam radiotherapy has better tissue-sparing for smaller σ/ctc, corresponding to larger peak-to-valley dose ratios PVDR, with the best effect for σ/ctc < 0.032. However, even quite large σ/ctc (e.g. σ/ctc = 0.23 or 0.31, i.e. PVDR = 10 or 2.7) show less acute side effects than a homogeneous dose distribution. This suggests that proton minibeam therapy spares healthy tissue not only in the skin but even for dose distributions appearing in deeper layers close to the tumor enhancing its benefits for clinical proton therapy.

## Introduction

Radiotherapy with highly energetic protons, light or heavy ions is one of the strongest growing fields in cancer therapy. The inherent physical advantages such as a limited range as well as the increasing dose deposition with depth (Bragg curve) are highly attractive for oncologists. The integral energy deposited in the healthy tissue, which causes limiting side effects, is reduced in comparison to x-ray therapy. The biological effectiveness of highly energetic protons is similar to photons (RBE ~ 1.1) [1], which allowed for the transfer of clinical experience of radiologists over the last decades to the treatment with protons. Although cancer treatment with protons and ions is beneficial compared to photons, side effects are still the limiting factor for the applied dose.

**Fig 1:**
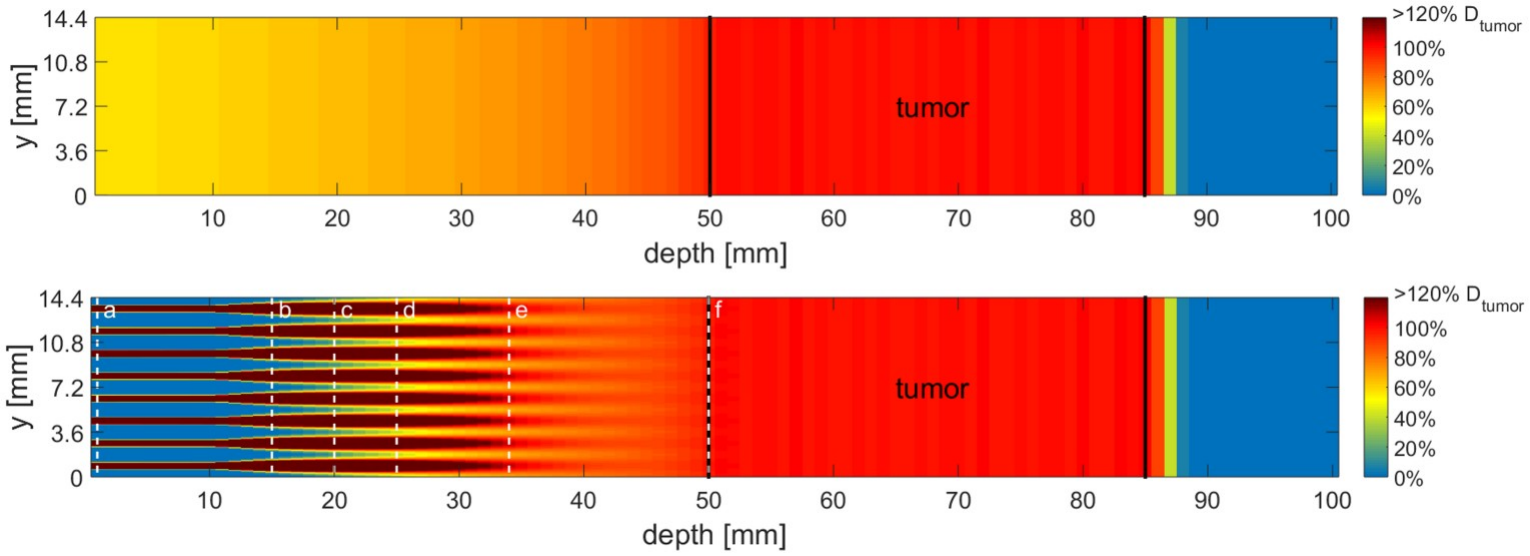
A side cut of the calculated dose distribution of a conventional proton irradiation and a proton minibeam irradiation for a tumor in 5–8.5 cm depth. The mean dose for both scenarios is the same in every depth, however, the distribution of the dose varies strongly. The dose is color-coded with a cut-off at 120% of the desired tumor dose D_tumor_. The black lines indicate the beginning and the end of the tumor. The dashed white lines labeled with a–f show the location where calculated minibeam sizes are the same as in the experimental setup with ~95, 199, 306, 411, 561 and 883 μm, respectively.

A novel technique, unidirectional proton minibeam radiotherapy, was recently introduced by Zlobinskaya et al. [2] and also mentioned by Prezado et al. [3]: Sub-millimeter sized pencil or planar proton beams, called proton minibeams, are applied in a pattern that covers the tumor volume laterally with center-to-center-distances (ctc) in the millimeter range. Due to small-angle scattering of the protons in the traversed tissue, minibeams increase in size with depth. By adjusting the ctc-distances such that the beams overlap at the proximal end of the target volume, a homogeneous irradiation of the tumor can be ensured for any tumor thickness [4, 5]. A calculated dose distribution for both, unidirectional proton minibeams and standard proton therapy is shown in Fig 1 for illustrating the differences in dose distributions. The calculations were performed according to our theoretical work of 2017 [4] and assume a tumor in a water phantom. Idealized Gaussian beam shapes with depth-dependent beam sizes are taken from LAP-CERR [6, 7]. The tumor dimensions were chosen such that the calculations fit the experimental setup of this work.

While the modulated dose distribution (peak and valley pattern) in spatial fractionation reduces side effects in the entrance channel [2, 5, 8, 9], the tumor control probability is high due to its homogeneous irradiation. This unidirectional minibeam application gives the opportunity to either reduce side effects in the healthy tissue, while maintaining a similar tumor control level as in conventional therapy or even enhance the chance of cure by increasing the tumor dose, such that the side effects remain similar to conventional treatments. Besides, a substantial reduction of the total number of applied fractions in time comes into reach.

The first proof of principle experiment in a mouse ear model has shown that acute side effects could be completely avoided by spatial fractionation if 180 μm squared minibeams are applied in a grid of 1.8 mm ctc-distance and a mean dose of 60 Gy (peak dose: 6000 Gy). In contrast, a homogeneous field irradiated with the same number of protons, hence the same mean dose of 60 Gy, led to severe radiation toxicities [5].

In another study, rat brains were irradiated with an average dose of 25 Gy and planar minibeam sizes of 400 μm in a ctc-distance of 3.2 mm [9]. While the homogenously irradiated fields showed substantial brain damage and severe skin reactions, the rats irradiated with proton minibeams showed significantly reduced brain damage and no skin damage in 7 out of 8 rats [9]. The follow-up study with similar beam parameters irradiated the rat brains with gliomas [10]. An even enhanced tumor control was found for the group treated with proton minibeams although a slight dose heterogeneity was still maintained in the tumor volume (PVDR ~ 1.2) [10].

Despite the tremendous amount of sparing potential which was demonstrated by the mentioned experiments [5, 9], there are still open questions remaining to be answered before the full potential of proton minibeam radiotherapy can be exploited. In addition to the mean dose, the main parameters in spatial fractionation are the beam size σ of an assumed Gaussian dose distribution and the ctc distance of the beams. The σ/ctc ratio describes the dose valleys and peaks relatively to a homogeneous dose distribution of the same mean dose, as does the peak-to-valley dose ratio PVDR. The ctc-distance depends only on tumor depth and size to ensure homogeneous tumor dose coverage [4]. However, the proton minibeam sizes depend on the initial beam size and the lateral spread of the beam by multiple Coulomb scattering which increases with depth. Thus, it is essential to measure how side effects depend on dose distributions resulting from different beam sizes and ctc-distances (i.e. σ/ctc ratio). In a previous study, single x-ray beams of different sizes from 0.5 mm to 6 mm (FWHM) were applied with a maximum dose of 60 Gy to the ears of BALB/c mice. The results showed that only very small skin reactions occurred for beam diameters smaller than or equal to 2 mm FWHM while the toxicities strongly increased for larger beams [11]. The single-beam study showed the principal limit of pencil beam sizes below which tissue-sparing by spatial fractionation can be obtained. Since the RBE of highly energetic protons is close to one [1] the results can be directly applied as the upper limit of proton pencil minibeam sizes for spatially fractionated dose schemes.

When applying minibeams in a grid or planar pattern, dose distributions of adjacent minibeams overlap and the tissue-sparing effect is reduced due to fewer healthy cells surrounding the irradiated tissue. As long as the relevant beam sizes are much smaller than 1 mm, as given from the single-beam experiment, the only parameter that remains determining side effects is the σ/ctc ratio. To study the interplay of proton pencil minibeams in a grid pattern, the established BALB/c mouse ear model [5, 11, 12] was used. Proton pencil minibeams of different sizes with fixed ctc distances of 1.8 mm and the constant mean dose of 60 Gy were used for irradiation. The pencil minibeam sizes varied from σ = 95 μm to σ = 883 μm (standard deviation), corresponding to σ/ctc ratios between 0.05 and 0.5. The minibeam pattern with the largest beam size (σ/ctc ~ 0.5) is similar to a homogeneous field. The side effects appearing after irradiation were monitored for 90 days after irradiation. The results provide an insight into the sparing effect of different dose distributions of minibeam irradiations as they could be applied on the skin or as they occur in depth due to the lateral spread of the minibeams. Also, cell survival calculations were made as a first-order approximation to get a deeper understanding of the mechanistic effects involved in the sparing effect.

## Materials and methods

### Animal model and ethical approval

To investigate the normal tissue acute side effects of proton pencil minibeam irradiation, an animal model without a tumor was chosen. The ears of BALB/c mice (albino stem) were defined as target structure since the expected acute side effects such as reddening and ear swelling are easy to observe and to measure. Moreover, the thin ears (~ 200–250 μm) allowed for a irradiation with 20 MeV protons and a precise dose application. The linear energy transfer (LET ~ 2.7 keV/μm) is nearly constant within the whole ear and the traversed protons are detected and counted behind the ear. The model was already used in previous studies [5, 11, 12] which allows for comparison of the results.

The female BALB/c mice (Charles River Laboratories, Sulzfeld, Germany) were 8–12 weeks old and had ad libitum access to food and water. The animal facility was temperature regulated and mice were exposed to a 12-hour light/dark cycle. The experiment was approved by the District Government of Upper Bavaria and followed the animal welfare and ethical guidelines of our institutions. A total number of 56 mice were used for the study.

### Irradiation conditions for the proton study

The right ears of BALB/c mice were irradiated with a minibeam pattern, which consisted out of 4x4 beams with ctc distances of 1.8 mm, as in previous studies [4, 11]. This ctc is suitable for a tumor in 5–8.5 cm depth as illustrated in Fig 1 and the number of beams was the largest fitting on the mouse ears and is–in therapy—adapted to the lateral tumor dimensions. The number of protons (~ 4.58 × 10^8^) within the single beams was kept constant, such that a mean dose of 60 Gy was applied over the irradiated area (~ 7.2 × 7.2 mm^2^; similar to the previous study of Girst et al. [5]).

Irradiation with 20 MeV protons was carried out at the Munich ion microprobe SNAKE of the 14 MV Munich tandem accelerator. A specially developed setup allows for biological experiments with cells, tissues and animals [5, 13–15]. An aluminum, temperature-controlled holder enabled the irradiation of the right ear of the mice. The 20 MeV protons with a range of ~ 4.6 mm and a linear energy transfer (LET) of ~ 2.7 keV/μm traversed the ear and finally hit a scintillator-photomultiplier detector for particle counting. The irradiation time (max ~ 30 min) was limited by a maximum anesthesia of 45 minutes and therefore required a particle count rate of 5 MHz. The resulting dead times of detector and detection electronics (~ 20%) were corrected using radiochromic EBT3 films (GafChromic^™^, Ashland, US). A radiochromic film was irradiated with a low particle count rate and then compared to an irradiated film with a high particle count rate. Since the counted number of protons was fixed, the dead time could be determined by the darkening ratio of both films. The protons were focused to a micrometer spot and subsequently scattered in a 200 μm thick aluminum layer that covered the exit nozzle of the microprobe. The minibeam size could be controlled by adjusting the distance of the ears from the aluminum.

Seven groups each consisting of 8 BALB/c mice were exposed to a single fraction minibeam irradiation and classified by the applied σ/ctc ratios of 0.053, 0.11, 0.17, 0.23, 0.31, 0.49 (corresponding beam sizes in standard deviation σ: 95, 199, 306, 411, 561, 883 μm, respectively). The desired beam sizes were classified by the expected valley doses of 0, 0, > 0, ~15, ~30 and 60 Gy. One group was sham irradiated as a control. All mice were monitored in intervals of 1–4 days dependent on the reaction for a 90-day follow-up period.

The mice were anaesthetized for the irradiation by intraperitoneally injected medetomidine (0.5 mg/kg), midazolam (0.5 mg/kg) and fentanyl (0.05 mg/kg). After a maximum of 45 minutes of anesthesia, the antagonist atipamezole (2.5 mg/kg), flumazenil (0.5 mg/kg) and naloxone (1.2 mg/kg) was administered subcutaneously.

### Ear thickness measurements

An electronic external measuring gauge (C1X079, Kröplin GmbH, Schlüchtern, Germany) was used to measure the thickness of both ears for the period of 90 days after irradiation. The measuring contacts of the gauge were 6 mm in diameter. Every ear was measured thrice per time point at the center of the ear. The measuring intervals were in between 1–4 days in dependence of the skin reaction.

### Skin reaction scoring

The irradiation resulted in skin reactions such as erythema (Score A) and desquamation (Score B). Both were visually scored by the four-eyes principle and summed up to a total skin score (Table 1). The scoring was performed at the same time points as the thickness measurements. The scoring accuracy was estimated as 0.5.

**Table 1:** Skin response score table.

Erythema	Scale	Desquamation	Scale
no	0	no	0
mild	0.5	dry	1
definite	1.5	crust formation	2
severe	3	moist	3

Erythema and desquamation scale are added together to obtain a total skin score (table adapted from Girst et al. [5]).

### Calculation of clonogenic cell survival in epidermal keratinocytes

The survival of cells in the ears after irradiation contributes to the measurable ear reactions such as desquamation and ear swelling. One of the main responsible cell types for the acute skin reaction after irradiation are keratinocytes [12, 16]. Hence, the linear-quadratic model was applied to the irradiated dose distributions of the six different minibeam sizes, using the corresponding α = 0.2 Gy^-1^ and β = 0.06 Gy^-2^ values for keratinocytes according to Parkinson et al. [17]. Ideal Gaussian dose distributions of standard deviation σ are taken for the minibeams that are placed on a quadratic grid of center-to-center distances ctc = 1.8 mm. The dose per minibeam is chosen such that a mean dose of 60 Gy is calculated for an infinite irradiation field. Within this approximation, the clonogenic cell survival depends only on the ratio σ/ctc. For each σ/ctc, the physical doses are translated into clonogenic cell survival via the linear-quadratic model and subsequently the mean within a unit cell of the pattern is determined similar to our theoretical study [5]. The effects of the high doses within the beams are overestimated using the linear-quadratic model, which is only accurate up to ~ 10 Gy. However, they differ only slightly in their absolute values if a linear extended model is used and can be neglected if only the percentage scale is taken into consideration as shown by Sammer et al. [5].

## Results

### Dose distributions for proton pencil minibeams and size verification

**Fig 2:**
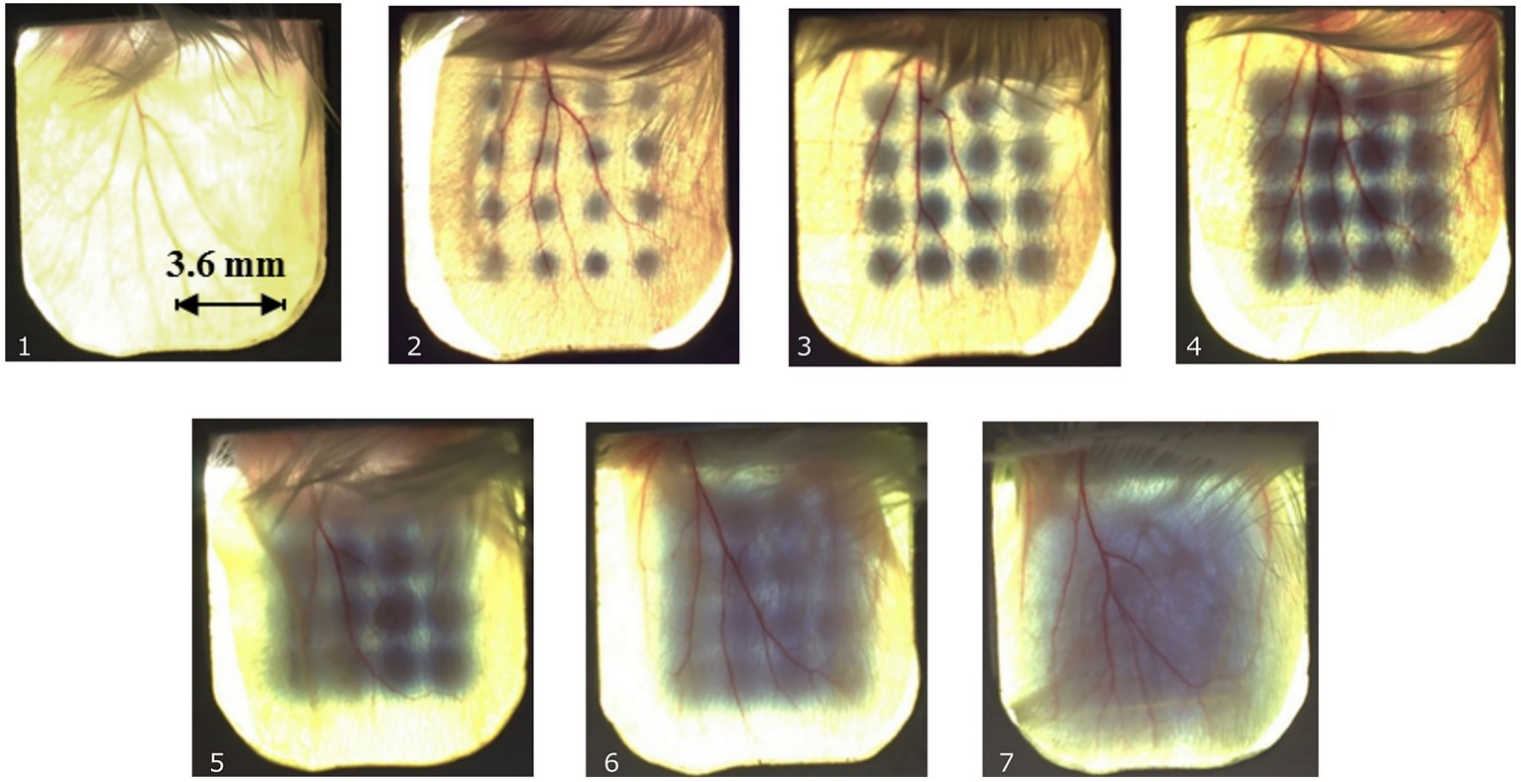
Gafchromic films mounted behind mouse ears show the irradiation pattern for non-irradiated ears (1) and irradiations with σ/ctc-ratios of 0.053 (2), 0.11 (3), 0.17 (4), 0.23 (5), 0.31 (6) and 0.49 (7) at ctc = 1.8 mm each. (7) corresponds to a homogeneous dose distribution. Owing to the limited sensitivity range of the films, no absolute dose values or minibeam sizes can be extracted from these images.

**Fig 3:**
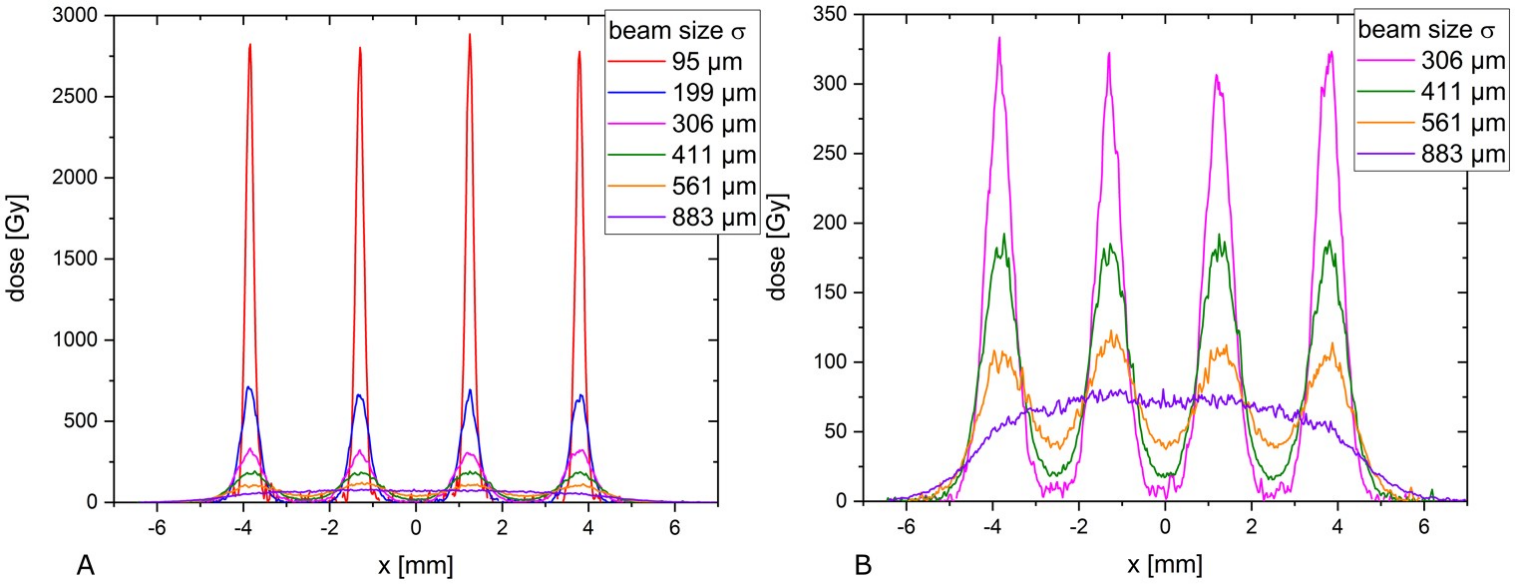
A) Measured dose profiles via radiochromic film irradiation extrapolated to an average dose of 60 Gy. B) The dose profiles of the largest four beam sizes are shown on an enlarged dose scale. All profiles were cut diagonally to the pattern to show the absolute minimum and maximum dose, hence the shown ctc distances are increased by the factor $\sqrt{2}$.

Fig 2 shows a photograph of one mouse ear from each group after irradiation with a mean dose of 60 Gy. A Gafchromic EBT3 film was placed behind the ear to verify the irradiation and visualize the different minibeam sizes. The applied 60 Gy mean dose was larger than the sensitivity range of the Gafchromic films and the shown films were therefore unsuitable for absolute dose verification. The absolute mean dose was measured by particle counting as (60 ± 3) Gy. The dose uncertainties result from the radiochromic dosimetry which was necessary to correct for the dead times of the proton detection. The dose profiles and beam sizes were analyzed by additionally irradiated Gafchromic EBT3 films where doses were adjusted such that they matched the sensitivity range. The measured beam sizes, i.e. standard deviations were obtained from fitting a Gaussian distribution over the profile of a single irradiated beam for each beam size (cf. Table 2). Beam size uncertainties result from two independent measurements of the beam sizes. Fig 3 shows the dose modulation differences, which vary from peak-to-valley dose ratios PVDR > 540 to PVDR ~ 1.1 for the smallest to the largest beams, respectively.

**Table 2:** Measured beam sizes (i.e. standard deviations).

Measured beam sizes σ [μm]	95.3 ± 1.4	198.6 ± 1.7	305.7 ± 2.5	411.0 ± 2.1	561 ± 4	883 ± 5
PVDR	> 540	> 132	47 ± 20	10.1 ± 0.9	2.69 ± 0.19	1.11 ± 0.10
σ/ctc	0.053	0.110	0.170	0.228	0.312	0.491

Beam sizes were measured twice with a Gafchromic film placed at the corresponding ear positions. The PVDR was extracted from the profile cuts. The PVDR values of the pattern with the two smallest beam sizes can just be given as a lower limit since the valley doses are lower than the noise level of the Gafchromic film. The given uncertainties arise from the Gaussian propagation of the determination of the maxima and minima to calculate the PVDR. The σ/ctc values are calculated as the corresponding beam size σ divided by the center-to-center distance (ctc = 1.8 mm).

### Skin response scoring

**Fig 4:**
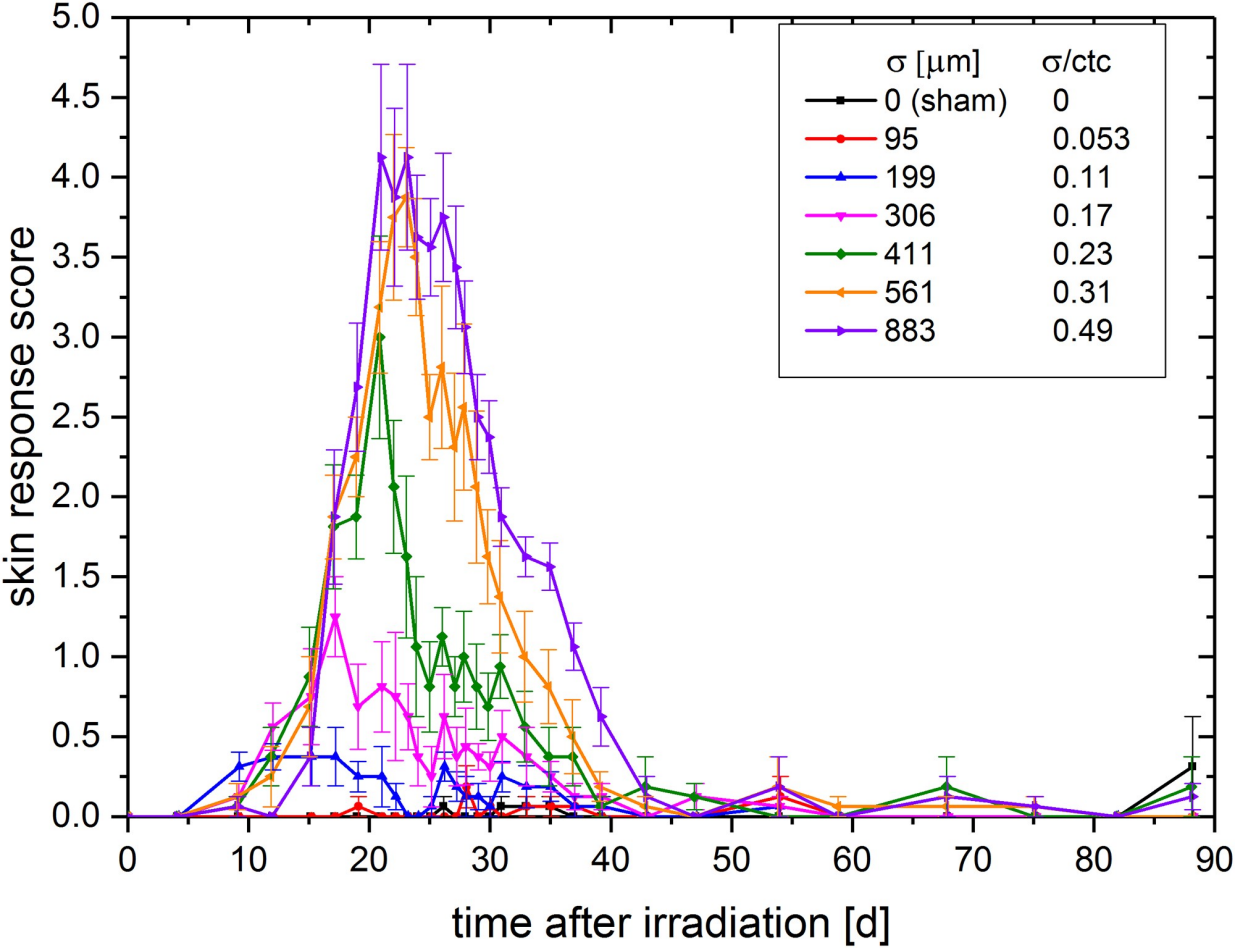
Mean score over monitoring time (sum of desquamation and erythema score ± SEM).

The skin response was scored according to the scores of Table 1 in intervals of 1–4 days dependent on the severity of the acute skin response for 90 days post-irradiation. The skin response score, defined as the sum of erythema and desquamation, is shown in Fig 4. The error bars result from the statistical errors in addition to an estimated systematic error of 0.5.

The score of the mouse group irradiated with the smallest minibeams (σ/ctc = 0.053, red line in Fig 4) was not distinguishable from the sham score (black line) during the 90-day monitoring period (p > 0.15). For all groups irradiated with σ/ctc ratios ≥ 0.11 a clear skin response was observable (p < 0.05). However, the skin response becomes more severe with increasing σ/ctc. The strongest overall reaction was obtained for the 0.49 σ/ctc ratio, which corresponds to a homogeneously irradiated field. No skin reactions were found anymore for any group later than ~ 45 days after irradiation. The temporal progression, i.e. onset, fall off and maximum of the reaction started earlier for smaller beam sizes and therefore correlated with higher peak doses.

### Measurement of ear thickness

**Fig 5:**
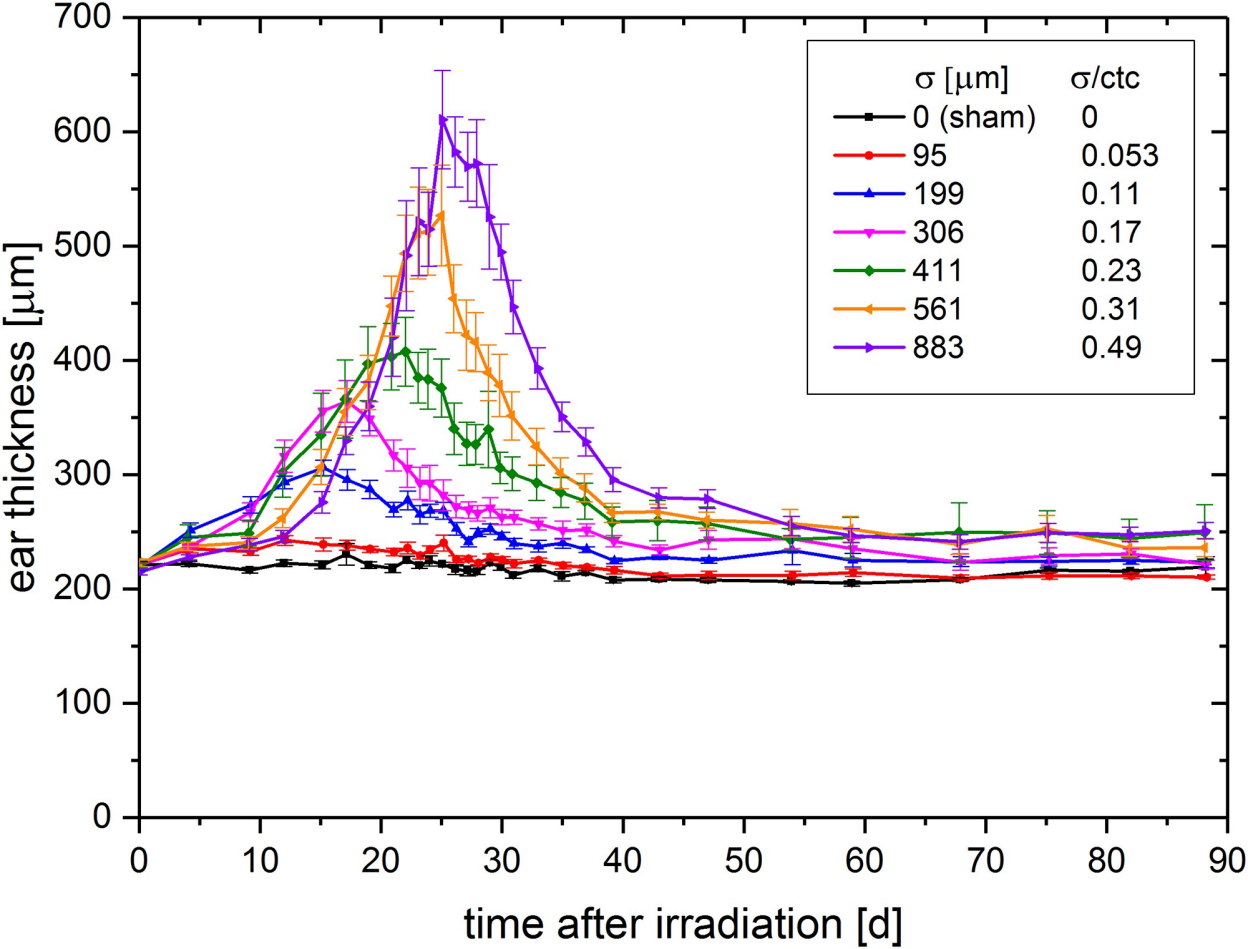
Mean ear thickness (± SEM) over time after irradiation.

The measurement of the ear swelling was conducted at the same time points as the skin response scoring. The mean ear thickness over the monitoring time of 90 days is shown in Fig 5.

There is a strong correlation between ear swelling and applied beam size (p<0.01). While the 0.053 σ/ctc ratio induced just a little ear swelling compared to the sham irradiated control group, the thickness increased strongly with increasing beam sizes. The homogeneously irradiated field with σ/ctc = 0.49 induced the strongest swelling to a maximum ear thickness of about 610 μm, hence about a 3-fold ear swelling (initial ear thickness ~ 200 μm; p<0.01). The temporal progression of the swelling curve confirms the observed skin score data with a trend towards earlier onset and maximum for the smaller σ/ctc ratios. The ear thickness of time points later than 60 days after irradiation reaches a steady state. This is similar for the skin response scoring, where no significant visible reaction is scored for time points later than 45 days after irradiation. However, the irradiation groups σ/ctc > 0.11 tend towards a slightly increased ear thickness up to the end of the experimental observation time, with even thicker ears (swelling ~ 30–40 μm) for larger irradiated σ/ctc ratios (> 0.23). This persisting ear thickening might indicate long term side effects like fibrosis but needs to be clarified in further studies.

**Fig 6:**
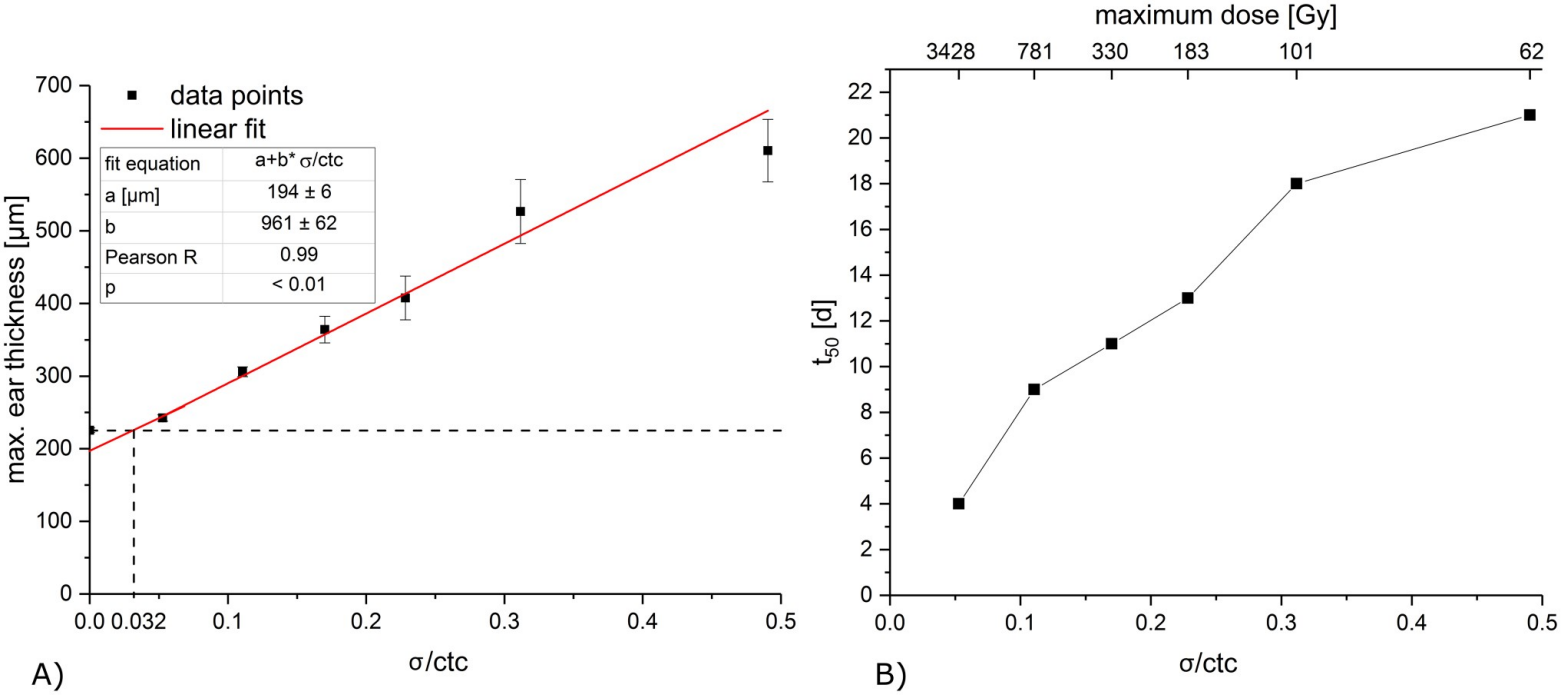
A) Maximum ear thickness over beam size σ to ctc ratio σ/ctc. The red line corresponds to a linear fit (R = 0.99). The dashed lines mark the ear thickness of the control ear and the intersection point of control ear thickness and linear fit. B) Timepoint t_50_ of half the maximum ear swelling over σ/ctc. The corresponding maximum doses are shown on the top x-axis.

If the maximum ear thickness (the maximum appears at different time points) is plotted over σ/ctc as shown in Fig 6A, a linear correlation between maximum ear thickness and σ/ctc is obtained. The control group was excluded for the fit since no radiation-induced ear swelling appears in the untreated ears and would, therefore, falsify the linear effect observed.

A point of intersection was found at σ_min_/ctc ~ 0.032 between the fit and the minimum thickness of the mouse ear (control t_0_ = 225 μm). This represents the σ/ctc below which no ear swelling can be detected and corresponds to a minibeam size of σ ~ 58 μm for the utilized ctc of 1.8 mm. The skin responses and thickening reactions show some interesting details in their time courses. While the maximum reactions are reduced for smaller beam sizes, the start of the reactions begins earlier. Thus, the reactions of the smaller minibeam sizes are even slightly enhanced at the first 10 to 20 days compared to the close to homogeneous irradiations. A monotonic increase is observed between the time point of half maximum ear swelling t_50_ and σ/ctc as shown in Fig 6B. The time shift towards earlier time points for smaller σ/ctc and thus higher maximum doses might indicate an influence of different cell death pathways for the high dose irradiated cells within the ears.

### Clonogenic cell survival calculation

**Fig 7:**
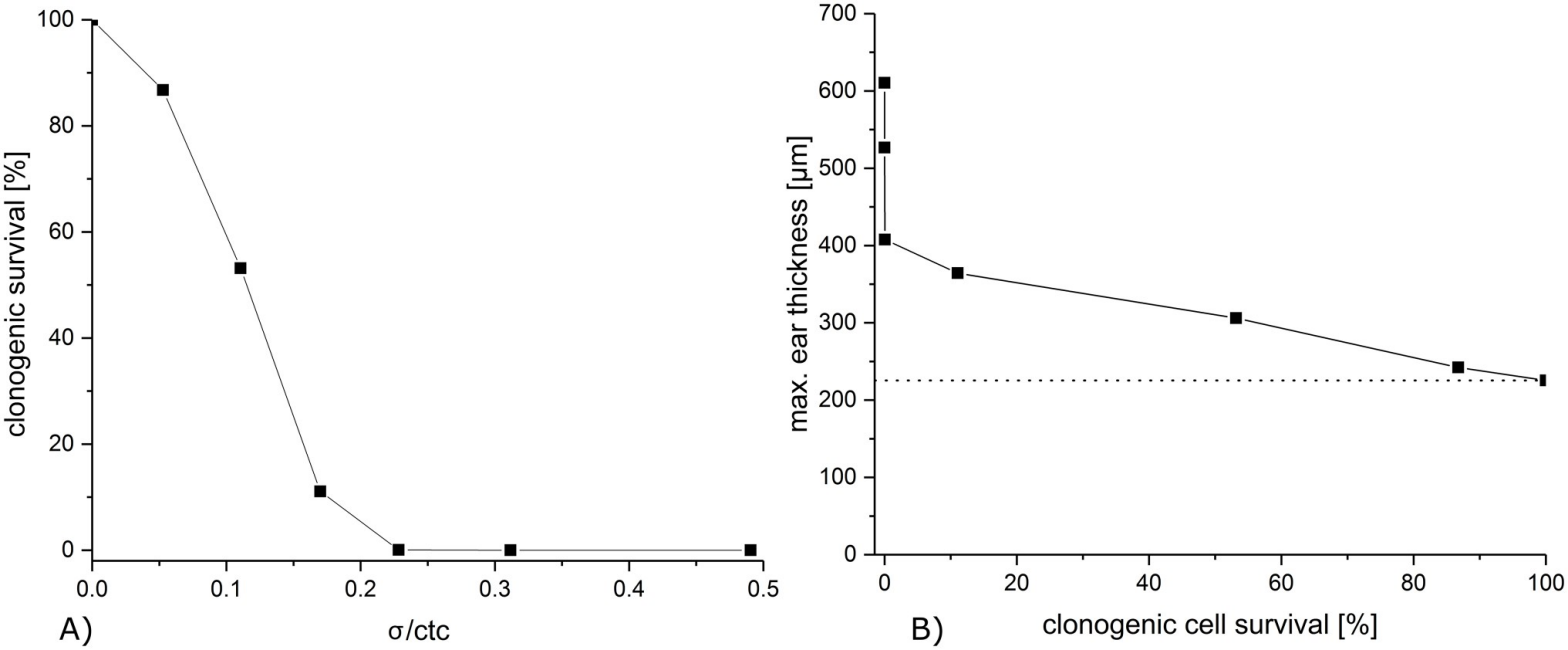
A) Clonogenic cell survival of mouse keratinocytes in dependence of the σ/ctc ratios. B) Maximum ear thickness over the calculated clonogenic cell survival of the corresponding σ/ctc. The dotted line marks the max. ear thickness of the unirradiated group.

The clonogenic cell survival within minibeam irradiated areas is calculated as a first-order approach to get a deeper understanding of the observed reactions. The calculated clonogenic cell survival is plotted over σ/ctc in Fig 7A for a 60 Gy mean dose. For σ/ctc greater than 0.2, there is less than 5% clonogenic cell survival with a sharp decrease for even bigger σ/ctc while the survival rate is larger than 90% for σ/ctc ratios smaller than 0.1 due to the spatially fractionated sparing. Although the clonogenic cell survival remains the same for all beam sizes as long as the σ/ctc and the mean dose stay the same, the radiation responses may still change depending on the absolute size of the minibeams due to the repair mechanisms of the tissue. A restriction may be given from the single-beam experiments in which a total diameter of 2 mm was assigned as an upper limit for the occurrence of only mild skin reactions [11].

The measured maximum acute radiation toxicity, represented by the maximum mouse-ear thickness, is plotted versus the calculated clonogenic cell survival for the corresponding σ/ctc in Fig 7B. While the maximum ear thickness increases just slowly with decreasing cell survival, a close to zero cell survival does not show a saturation effect. The three biggest σ/ctc (> 0.23), which all result in < 1% cell survival, show very different responses with the strongest response for the σ/ctc ~ 0.5, equivalent to a homogeneous irradiation.

Thus, the number of proliferating cells may not be the only parameter that determines the radiation responses. Cells irradiated with hundreds of Gy may not only be stopped from proliferating but may have a higher probability of necrosis or apoptosis, which in turn alters the tissue repair. Furthermore, migration of viable cells adjacent to the minibeams needs to be taken into account for tissue repair, which is again dependent on the size of the radiation-injured area. However, detailed models are missing to calculate the cell death pathway fractions for different doses as they appear in the inhomogeneous dose distributions of spatial fractionation.

## Discussion

The scheme of a unidirectional proton minibeam therapy as discussed in [2, 4, 5] allows a homogeneous dose coverage of the tumor while profiting from spatial dose fractionation in the healthy tissue. A similar unidirectional approach by applying x-ray micro- or minibeams to cover the tumor homogeneously is not possible since dose is also deposited distally to the tumor. Heavy ions (e.g. He-ions, boron, carbon or oxygen ions) are also suitable for minibeam therapy just as protons, but the beams have to be initially smaller since the lower scattering of heavier ions requires smaller ctc distances to form a homogenous tumor dose, while also sparing healthy tissue.

Unidirectional proton or heavy ion minibeam therapy is technically less demanding than using interlacing minibeams from various directions. Interlacing particle minibeams would have even larger sparing potential since spatial fractionation effects can be maintained close to the tumor. Particle minibeams from more directions could be interlaced due to the limited range of the particle beams enabling interlacing even from opposite directions [2, 18, 19]. Nevertheless, interlacing beams are technically more demanding to fulfill due to the necessary precision of beam adjustments to obtain dose homogeneity in the tumor. Besides, interlacing micro- or minibeams would suffer much more from organ and/or tumor movement.

In either case, tumor control can be expected to be the same as in conventional radiotherapy when the same homogeneous dose distribution is applied within the tumor. This might be an advantage compared to the proposed x-ray micro- and minibeam approaches which retain an inhomogeneous dose distribution inside the tumor [20, 21]. Even though the results of the animal studies in terms of tissue-sparing of healthy tissues are very promising for future implementation into clinics, more detailed investigations need to be carried out in terms of beam sizes, ctc distances of the beams, mean doses applied and the dependence on penetrated tissues by the minibeams. Afterwards, the sparing effects can be predicted and the full advantage of proton minibeam radiotherapy can be exploited.

The present study was carried out to compare side effects of proton pencil minibeam irradiations of different pencil beam sizes for a given grid pattern with 1.8 mm ctc distances in an *in-vivo* mouse ear model. The animal model allowed for a radiation response study of proton pencil minibeams in a living mammalian organism with similar radiation responses as in human skin, even though the doses necessary to induce similar side effects in humans might vary. The direct comparability of the different irradiations was ensured by keeping the mean dose (60 Gy) and the ctc distances (1.8 mm) constant. Only the beam sizes of the proton pencil minibeams were varied from approximated Gaussian σ between 95 μm and 883 μm (σ/ctc ratios between 0.053 and 0.49). The experimental setup allows for different perspectives and interpretations of the results.

### Determination of beam size to obtain full tissue-sparing by spatial fractionation

The results (Figs 4 and 6) show the dependency of the skin reaction on the σ/ctc ratios for a mean dose of 60 Gy. By applying different beam sizes to the skin of the mice ears for a square grid of 1.8 mm ctc distances, the maximum σ/ctc ratio for a proton pencil minibeam radiotherapy which would result in no side effects was extrapolated to σ/ctc = 0.032. The results show that larger σ/ctc ratios are still beneficial compared to homogeneous irradiations, but side effects increase with increasing σ/ctc-ratios. Considering a clinical irradiation scenario, beam sizes should be chosen smaller than given through the limit σ < 0.032 · ctc. The ctc distances are determined by the size and the location of the tumor to realize a homogeneous dose distribution in the tumor [4, 5]. The closer the tumor is to the skin, the smaller the ctc distances have to be chosen to fulfill the homogeneity constraints for the tumor. This leads to even smaller initial beam sizes to obtain negligible radiation responses. Smaller proton beams are harder to prepare or cannot even deliver the beam currents for an efficient proton pencil minibeam radiotherapy treatment. The presented study shows that larger beams with σ > 0.032 ∙ ctc show some, but still less skin reactions than a homogeneous irradiation. Hence, when beam sizes are limited through technical constraints, a compromise of the tissue-sparing potential may still be acceptable and, most important, beneficial for the patients.

Since the critical σ/ctc ratio below which no side effects occur is determined to σ/ctc = 0.032, the critical beam size is σ = 60 μm (FWHM ~ 140 μm) for the given ctc = 1.8 mm. In a previous experiment, single x-ray pencil beams were applied to the same mouse ear model and no side effects were found for sharply shaped beams up to 1 mm in diameter and a 60 Gy plateau dose [11]. The appearing difference may be due to the reduced number of proliferating cells in the close neighborhood of the minibeams within the grid pattern caused by the overlapping dose distributions. In addition, the number of apoptotic or necrotic cells, leading to a fast loss of the cells within the tissue, may be increased in the proton grid irradiation experiment since maximum doses exceeded the 60 Gy mean doses by factors (see Fig 3). This may lead to the faster but smaller reactions for the small minibeams (σ/ctc ratios: 0.11–0.23; σ: 199 μm to 411 μm; Figs 4 and 5). According to our theoretical study [4], typical ctc distances to treat a tumor in a human body are between 1–6 mm. Concluding from the mouse data, technical developments should ideally aim for σ = 32 μm. However, even σ/ctc ratios of ~ 0.1–0.15 induce only minor side effects corresponding to beam sizes of σ = 100–900 μm at the assumed ctc range.

### Dose distributions within a tumor irradiation scenario

Another perspective of the results is the interpretation of side effects for the different σ/ctc ratios as they appear for proton pencil minibeams on their way to the tumor in deeper-lying tissues (such as muscles or organs). The 1.8 mm ctc would be ideal to treat a target volume in ~ 5–8.5 cm depth (according to [4]). The necessary energies to irradiate such a target would be between 79 and 107 MeV. However, the relative biological effectiveness is RBE ~ 1.1 of both the used 20 MeV as well as the higher, clinical energies and does therefore barely influence the results. By increasing the 20 MeV proton beam sizes, the dose distributions are similar to those appearing in depth from the higher energetic protons in the healthy tissue due to the small-angle scattering of the protons. A tumor irradiation with proton minibeams is calculated and the dose distribution is shown in Fig 1. The corresponding depths to the applied dose distributions are marked in Fig 1 (white dashed lines) and the values are listed in Table 3.

**Table 3:** Corresponding depths to the irradiated σ/ctc-ratios and beam sizes for an exemplary tumor in 5–8.5 cm depth.

σ/ctc-ratio	0.053	0.11	0.17	0.23	0.31	0.49
Beam size σ [μm]	95	199	306	411	561	883
Depth d [cm]	0	1.5	2.0	2.5	3.4	5.0

The proton scattering data were taken from the database LAP-CERR [6, 7].

Therefore, all irradiated dose distributions can be considered as artificial cuts (perpendicular to the beam incidence) from deeper layers of a unidirectional proton pencil minibeam treatment. The group with the biggest σ/ctc ratio of 0.49 plays a particular role in this interpretation, as it represents the homogeneous dose distribution. From the unidirectional proton pencil minibeam treatment point of view, it is the dose distribution that appears in the target volume. For conventional proton therapy, however, it represents the dose distribution in each depth of a tumor treatment, including the whole entrance channel in healthy tissues. Of course, the mean dose would increase with depth similar to conventional proton radiotherapy due to the Bragg curve in a unidirectional proton minibeam treatment. However, the study was conducted to observe the geometrical influences of a spatially fractionated proton pencil minibeam radiotherapy on the healthy tissue response. Hence, it was necessary to keep the mean dose constant (60 Gy) to have a distinct outcome due to pure geometrical variations of the dose distributions rather than any additional influence of varying mean doses. Eventually, it was possible to compare the different minibeam irradiations, which represent the dose distributions of certain depths, to the corresponding conventional irradiation, all represented by the σ/ctc ratio = 0.49 group. Nevertheless, differences in proliferation, necrosis, migration and repair in normal tissue types other than skin might influence the absolute beam sizes required for certain tissue reactions and will have to be elaborated in future studies.

The presented study reveals that most damage is caused by the homogeneous irradiation as it appears close to and within the tumor. Furthermore, not only the smallest σ/ctc ratios, but also larger σ/ctc ratios were found beneficial regarding a skin reaction and compared to the homogeneous case. This result may hold for any minibeam treatment case as long as the minibeam sizes are small enough that radiation toxicities from single-beam irradiations also remain small. Total beam sizes smaller than 2 mm diameter for minor and less than 1 mm diameter for no skin reaction of single beams were obtained as upper limits for minibeam sizes [11]. The interaction of the beams in an irradiation grid depends only on the σ/ctc ratios as long as beam sizes are smaller than these single-beam limits. Thus, less side effects are expected in the whole entrance channel with increasing benefits for tissues closer to the surface for a proton pencil minibeam radiotherapy treatment, as long as the discussed single-beam limits are not exceeded.

## Conclusion

This study of skin reactions in a mouse ear model has shown a clear reduction of side effects after proton pencil minibeam irradiation compared to conventional homogeneous irradiation. The variations of the beam size while keeping dose and ctc distances constant allowed for a differentiated insight into the beneficial effects of spatially fractionated dose distributions with protons that appear in the skin as well as in deeper layers. The study confirmed that technical developments need to aim for minibeam sizes below 0.1 mm at best. However, it was observed that any spatial fractionation with submillimeter proton beams leads to reduced side effects and therefore could become an attractive option in clinical proton therapy to increase the therapeutic index.

## Supporting information

S1 Data All measured and scored mouse data. Every measurement was repeated thrice and the scoring was performed under four eyes principle.
(XLSX)
